# Is antibiotic treatment effective in the management of chronic low back pain with disc herniation? Study protocol for a randomised controlled trial

**DOI:** 10.1186/s13063-021-05728-1

**Published:** 2021-10-30

**Authors:** Donna M. Urquhart, Jeffrey V. Rosenfeld, Maurits van Tulder, Anita E. Wluka, Karin Leder, Allen C. Cheng, Andrew B. Forbes, Patrick Chan, Richard O’Sullivan, Susan Liew, Flavia M. Cicuttini

**Affiliations:** 1 Department of Epidemiology and Preventive Medicine, School of Public Health and Preventive Medicine, Monash University, Alfred Hospital, Melbourne, VIC 3004 Australia; 2 Department of Neurosurgery, Alfred Hospital, Melbourne, VIC 3004 Australia; 3 Department of Surgery, Central Clinical School, Monash University, Melbourne, VIC 3004 Australia; 4 Department of Human Movement Sciences, Faculty Behavioural and Movement Sciences, Vrije Universiteit Amsterdam, 1081 HV Amsterdam, The Netherlands; 5 MRI Department, Healthcare Imaging Services, Epworth Hospital, Richmond, VIC 3121 Australia; 6 Department of Orthopaedic Surgery, Alfred Hospital, Melbourne, VIC 3004 Australia

**Keywords:** Antibiotics, Low back pain, Disc herniation, Modic change, Randomised controlled trial

## Abstract

**Background:**

There has been immense interest and debate regarding the effectiveness of antibiotic treatment for chronic low back pain. Two randomised controlled trials have examined the efficacy of antibiotics for chronic low back pain with disc herniation and Modic changes, but have reported conflicting results. The aim of this double-blind, randomised, placebo-controlled trial is to determine the efficacy of antibiotic treatment in a broader patient subgroup of chronic low back pain with disc herniation and investigate whether the presence of Modic changes predicts response to antibiotic therapy.

**Methods:**

One hundred and seventy individuals with chronic low back pain will be recruited through hospital and private medical and allied health clinics; advertising in national, community and social media; and posting of flyers in community locations. They will be randomly allocated to receive either amoxicillin-clavulanate (500 mg/125 mg) twice per day for 90 days or placebo. The primary outcome measure of pain intensity will be assessed using the Low Back Pain Rating scale and a 100-mm visual analogue scale at 12 months. Secondary measures of self-reported low back disability and work absence and hindrance will also be examined, and an economic analysis will be conducted. Intention-to-treat analyses will be performed.

**Discussion:**

There is uncertainty about whether antibiotic treatment is effective for chronic low back pain and, if effective, which patient subgroup is most likely to respond. We will conduct a clinical trial to investigate the efficacy of antibiotics compared with placebo in individuals with chronic low back pain and a disc herniation. Our findings will provide high-quality evidence to assist in answering these questions.

**Trial registration:**

Australian New Zealand Clinical Trials Registry ACTRN12615000958583. Registered on 11 September 2015

**Supplementary Information:**

The online version contains supplementary material available at 10.1186/s13063-021-05728-1.

## Background

Low back pain is a major global health issue. It is not only the leading cause of disability worldwide [1], but evidence-based treatments are limited and only provide small to moderate benefits [2]. Recently, there has been enormous interest and controversy around a potential new treatment approach involving antibiotic therapy for a specific patient subgroup with chronic low back pain [3, 4]. The approach is based on the hypothesis that some individuals that sustain a disc herniation develop chronic low back pain due to a secondary, low-grade infection that develops in the disc [5]. This infection may result from a breach in the disc’s integrity allowing low virulent organisms that are commonly present on human skin to travel via the blood stream to the disc, resulting in infection, adjacent bone oedema (Modic change) and disabling back pain.

The first randomised controlled trial of antibiotic therapy by Albert et al. showed that amoxicillin-clavulanate (500/125mg) three times per day for 100 days was more effective than placebo for chronic low back pain with disc herniation and adjacent Modic type 1 changes (vertebral bone oedema) [6]. These findings were considered analogous to the discovery of *Helicobacter pylori* and the treatment of gastric ulcers, where a previously common, non-infectious condition was found to have an infective origin. While these results show promise for the prescribing of antibiotics for a specific patient subgroup with low back pain, commentaries in the BMJ highlight the importance of further research before a change in practice can be justified [3, 4]. More recently, a second clinical trial by Bråten et al. found that treatment with antibiotic therapy, amoxicillin (750 mg) 3 × per day for 3 months, did not provide a clinically important benefit in patients with chronic low back pain and Modic type 1 or 2 changes at the level of a previous disc herniation [7]. While these trials by Albert et al. and Bråten et al. provide contrasting results regarding the efficacy of antibiotic treatment for chronic low back pain, there are key differences in their methodology, including differences in the type of Modic changes examined and the type of antibiotic treatment prescribed, which may explain the disparity [6, 7].

We previously conducted a systematic review to comprehensively examine whether bacteria have a role in low back pain and to investigate the relationship between bacteria and Modic changes [8]. Our review found that bacteria are present in the spinal discs of people with back pain (undergoing spinal surgery), and there are a variety of bacteria present, including both *Cutibacterium acnes* (formerly known as *Propionibacterium acnes*) and *Coagulase-negative staphylococci*. We also found a relationship between the presence of bacteria and both low back pain with disc herniation and Modic changes associated with disc herniation. The findings of this systematic review and the previous clinical trials provide direction for future studies, including the need to further investigate the efficacy of a broad-spectrum antibiotic given the wide variety of bacteria identified, as well as the need to explore the defining clinical features of a patient subgroup, in particular disc herniation, which may benefit from antibiotic treatment.

Overall, while there is evidence for a role of bacteria in chronic low back pain, it remains unclear whether antibiotic treatment is effective for chronic low back pain and whether disc herniation with Modic changes represents a select target population, or whether all individuals with disc herniation may respond to antibiotics. We propose a double-blind, randomised, placebo-controlled trial to determine whether broad-spectrum antibiotic treatment (amoxicillin-clavulanate) is effective in reducing pain in individuals with chronic low back pain and disc herniation. Our secondary aims are to determine whether antibiotics are effective in reducing disability and work absence and hindrance and investigate whether the presence of Modic changes predicts those individuals more likely to respond to antibiotic therapy. A cost-effectiveness analysis will also be conducted. If found to be effective, the trial will provide high-quality evidence to support the use of antibiotics for chronic low back pain with disc herniation with or without Modic changes, a major area of unmet need. If we do not find amoxicillin-clavulanate to be effective, then our trial will provide strong evidence to consider it as an inappropriate approach in the management of chronic low back pain with disc herniation.

## Methods

### Study design

This study is a double-blind, randomised, placebo-controlled trial, with a two-arm, parallel group, superiority design. The trial was registered at the Australian New Zealand Clinical Trials Registry prior to recruitment (ACTRN12615000958583), and trial reporting will be guided by the Consolidated Standards of Reporting Trials (CONSORT) [9] and Standard Protocol Items: Recommendations for Interventional Trials (SPIRIT) [10] guidelines (see Additional file 1). Ethics approval has been obtained from the Alfred Hospital (526/14) and Monash University (CF15/1306 - 2015000623) Human Research Ethics Committees.

### Participants

A total of 170 individuals with chronic low back pain and lumbar disc herniation will be recruited through (i) general practitioners, medical specialists and allied health professionals; (ii) advertising on social media (including Facebook), and in community magazines and national newspapers; and (iii) posting of flyers in community locations such as shops, libraries and medical clinics. Written informed consent will be obtained from all participants by research staff trained in study procedures specific to this trial.

### Inclusion criteria

We will recruit male and female participants aged 18–60 years with chronic low back pain, which is defined as pain between the lower borders of the rib cage and the gluteal folds that has been present for greater than 3 months [11, 12] and with the presence of a disc herniation on magnetic resonance imaging (MRI).

### Exclusion criteria

Participants with any of the following will be excluded: (1) specific pathological entity, such as infection, metastasis, osteoporosis or fractures; (2) any contra-indication or allergy to antibiotic therapy; (3) antibiotic therapy in the past 3 months; (4) a compromised immune system; (5) osteomyelitis; (6) any kidney disease; (7) planned surgery in the next 6 months; (8) major co-existing illness which might confound the assessment of function; (9) another significant musculoskeletal condition; (10) pregnancy, planning or trying to become pregnant or breast feeding; or (11) inability to give informed consent, including individuals that are unable to read, speak or understand English.

### Randomisation and blinding

Randomisation will be performed by a computerised random number generator that will be held by an independent researcher not involved in other aspects of the trial. Allocation concealment will be ensured by the use of a central automated allocation procedure, with security in place to ensure allocation data cannot be accessed or influenced by any person.

The randomised controlled trial will be double-blinded, with both participants and investigators assessing outcomes blinded to treatment allocation. Allocation concealment and double blinding will be ensured by (1) the medications being dispensed by a Therapeutic Goods Administration licensed facility which specialises in product manufacturing of investigational products for the pharmaceutical industry, (2) the use of a placebo tablet that is identical in appearance and (3) questionnaires that are administered by research assistants blinded to group allocation.

### Intervention

Participants in the intervention arm will receive the antibiotic, amoxicillin-clavulanate (coamoxiclav; 500 mg/125 mg) tablets (Aspen Pharmacare Australia, NSW), two times per day for 90 days, and those in the control group will receive microcrystalline cellulose tablets, which will be identical in size, colour, coating and packaging to the active tablets. We selected amoxicillin-clavulanate for several reasons: (i) it is widely used in clinical practice; (ii) the initial clinical trial by Albert et al. found amoxicillin-clavulanate to have a beneficial effect on chronic low back pain with disc herniation and Modic changes type 1 [6]; (iii) our systematic review of biopsy studies showed that a variety of bacteria are present in the spinal disc and a broad-spectrum antibiotic, such as amoxicillin-clavulanate, is needed [8]; and (iv) there is evidence that it can penetrate the spinal discs [13]. All participants will be provided with usual care by their treating health practitioners.

### Study procedure

**Fig. 1:**
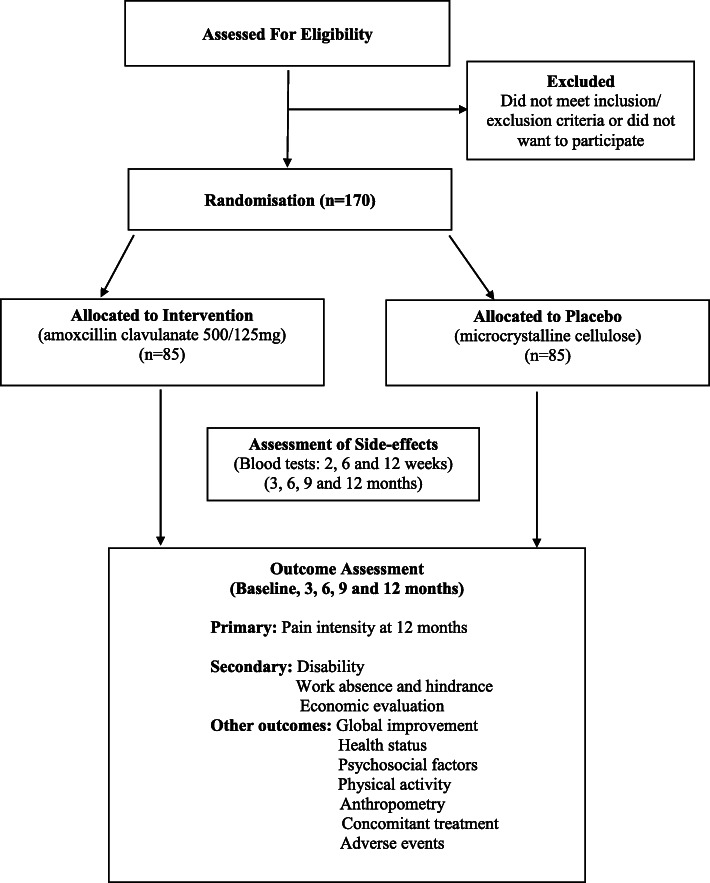
Trial flow diagram

The study procedures are presented in Fig. 1 and Table 1. Potential participants will be initially screened over the phone using a questionnaire to determine whether they meet the eligibility criteria. They will then attend an initial assessment at Monash University Department of Epidemiology and Preventive Medicine in Melbourne (State of Victoria, Australia) with the aim of obtaining informed consent and further examining the individual’s eligibility to participate in the trial. A full blood examination (FBE) and electrolyte and liver function tests will be performed to exclude conditions such as liver impairment or renal failure, and a c-reactive protein (CRP) blood test will be performed to detect inflammatory conditions such as osteomyelitis. A lumbar spine MRI will also be performed at baseline to determine whether a disc herniation is present and allow for the assessment of Modic changes. The trial will not involve collecting biological specimens for storage.

**Table 1:**
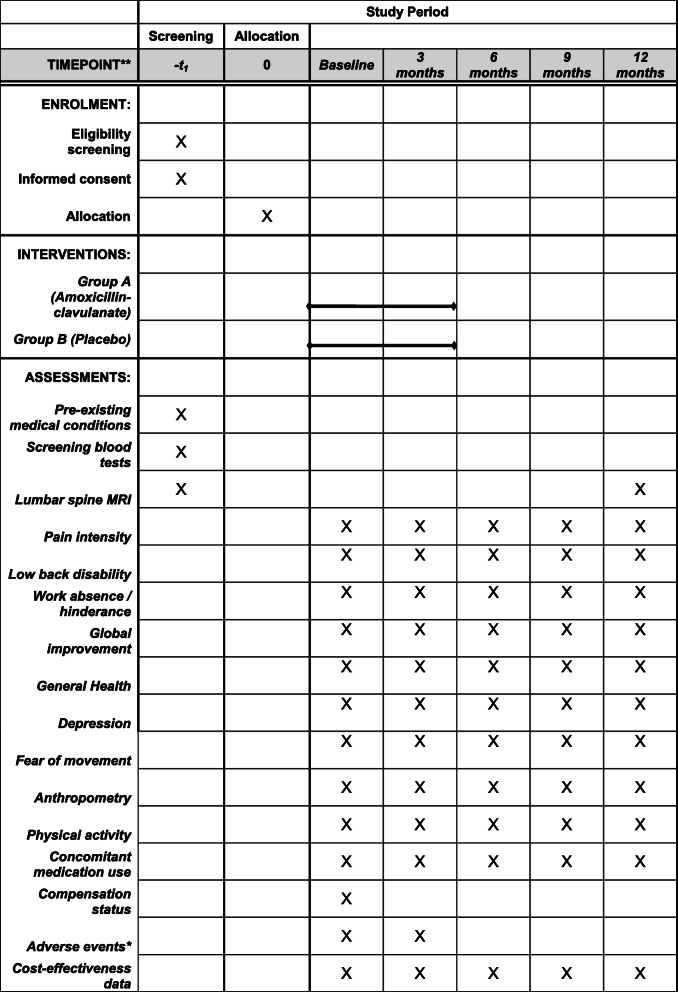
Schedule of enrolment, interventions and assessments

*Adverse events were assessed with a blood test at additional time points not presented in this table; including 2 and 6 weeks to monitor liver function and any other adverse events

Eligible participants will be randomised, asked to complete the baseline questionnaire and sent the first 3 months of amoxicillin-clavulanate or placebo, followed by the second 3 months half way through the trial. A blood test will be performed at 2, 6 and 12 weeks to monitor liver function and any other adverse events. Participants will also be requested to report any adverse events to the research staff spontaneously. Details of major adverse events and their relationship with study intervention will be recorded and reported to the ethics committees. If participants experience adverse events as a result of this research project, appropriate medical treatment will be provided to participants. All participants’ physician will be notified of their inclusion in the trial.

Participants will be contacted at 3, 6, 9 and 12 months to continue to monitor any side effects of the treatment and collect outcome data. A lumbar spine MRI will be conducted at 12 months to assess the presence of Modic changes. The same researchers, who are blinded to treatment allocation, will measure all clinical variables, administer questionnaires, monitor compliance and record adverse events. Unblinding will be allowed under certain circumstances, such as a participant’s doctor requiring their allocated intervention to ensure they receive the appropriate medical care. Compliance by trial medication will be assessed by pill count. Participants will not be paid for their participation in the trial, but they will be reimbursed for parking and transport costs.

### Outcome measures

The following primary and secondary self-report outcome measures will be provided by blinded research assistants at baseline and 3, 6, 9 and 12 months in the form of printed questionnaires sent by postal mail and/or electronic versions of the questionnaires emailed to participants.

#### Primary outcome measures: pain intensity

Our primary outcome measure will be pain intensity measured at 12 months using the Low Back Pain Rating Scale and a 100-mm visual analogue scale. The Low Back Pain Rating Scale is a valid measure of pain [14]. It consists of three 11-point box scales measuring current pain, the worst pain within the last 2 weeks and average pain within the last 2 weeks. These three scores are measured and averaged. The Low Back Pain Rating Scale was used in the previous trial by Albert et al. [6], enabling calculation of study sample size and comparison of results.

#### Secondary outcome measures: disability, work absenteeism and hindrance in work performance

Disability will be assessed using the Roland Morris Disability Questionnaire, which is a validated instrument designed to assess self-rated disability caused by low back pain [15]. We will examine absenteeism and hindrance in the performance of paid and unpaid work using The Short form Health and Labour questionnaire. This is a validated questionnaire used to collect data on productivity losses due to injury or sickness in individuals involved in paid or unpaid work [16].

##### Global improvement

A 6-point scale, ranging from ‘much worse’ to ‘completely recovered’ will be used to assess global improvement [17].

##### General health status

General health status will be measured using the EuroQol Instrument (EQ-5D-5L) [18].

##### Depression

Severity of mood symptoms will be assessed using the Beck Depression Inventory [19].

##### Fear of movement/(re)injury

Fear of movement/(re)injury will be examined using the 17-item Tampa scale [20].

### Potential explanatory factors

#### Anthropometry

Height (stadiometer), weight (electric scales) and body mass index (height/ weight^2^) will be measured at baseline.

#### Physical activity

The International Physical Activity Questionnaire short version [21] will be used to assess physical activity.

#### Concomitant medication use

The use of non-opioid analgesics and non-steroidal anti-inflammatory drugs will be allowed during the trial. The randomisation process is the most effective method for ensuring 2 groups are as similar as possible with respect to known confounders and unknown potential confounders including treatments. We will also collect data on the medication used and adjust for this in the analyses.

#### Compensation status

For each individual, we will record whether their back pain is associated with a compensation claim, and if so the nature of the claim, including the type, duration, items approved and associated costs.

#### Adverse events

We will monitor for adverse events through regular blood tests to assess participants’ kidney and liver function (2, 6 and 12 weeks), as well as asking participants a series of clinical questions, including whether they have seen a doctor, been to a hospital or experienced any new symptoms. Research staff will also request that participants spontaneously report adverse events to them if they occur. If an adverse event is reported, we will record the type, frequency and severity of the event, including whether it is considered to be unrelated or associated with the study drug.

### Data collection for economic evaluation

Cost data will be measured using cost questionnaires completed by participants at baseline and 3, 6, 9 and 12 months of follow-up. The questionnaires will refer to a previous period of not more than 3 months to prevent issues with recall bias. The cost questionnaires will include information on all health care utilisation due to chronic low back pain, such as the number and type of medical and allied health visits, imaging usage, amount and type of medication (using The Medication Quantification Scale [22]), other care/assistance (i.e. home-care assistance) and hours of absenteeism from paid and unpaid work. Unit costs of resources will be obtained from the Australian Pharmaceutical Benefits Scheme’s manual of costs. The Short form Health and Labour questionnaire will allow data on absenteeism from paid work, production losses without absenteeism from paid work and hindrance in the performance of paid and unpaid work to be collected [16]. The cost questionnaires and questionnaire data will be recorded during phone contact.

### Sample size calculation

#### Primary outcome: reduction in pain intensity

With 85 patients in each arm of the trial, there will be 90% power to detect a difference of 0.50 standard deviations in our primary outcome measure of pain. For the Low Back Pain rating scale, and assuming a between-person *SD* of 3.0 [6], a difference of 1.5 units can be detected with 90% power. A clinically significant difference in pain intensity is regarded as being at least 2 units [23].

#### Presence of Modic changes

For the secondary aim of assessing whether the effect of antibiotics compared to placebo is dependent on the presence of Modic changes, the relevant power calculation is that for detecting an interaction between antibiotic intervention and Modic status. We anticipate half the patients will demonstrate Modic changes at baseline, and assuming a between-person SD of 3.0 in LBP rating scale [6], 85 patients per randomised arm will have 80% power to detect an interaction effect of size 2.6, meaning that the effect of antibiotics in patients with Modic changes is 2.6 units higher than in patients without Modic changes. The value of 2.6 was observed in Albert et al. [6], and therefore, there is sufficient power to detect a large antibiotic effect in the Modic changes subgroup compared with little effect, if any, in other patients. If there is a significant interaction effect between antibiotic effect and Modic changes, this will provide evidence to support the hypothesis that infection is important in the underlying pathogenesis in this subgroup of patients with chronic low back pain and disc herniation. All analyses will adjust for the baseline value of the relevant outcome variable, and this will further increase the power by an amount depending on the size of the baseline to follow-up correlation in the outcome.

### Statistical analysis

Summary statistics comparing randomised arms at baseline will be tabulated. Intention-to-treat analyses of primary and secondary continuous outcomes will be performed by linear regression adjusting for the baseline of the outcome variable. Logistic regression will be performed for binary outcomes. Adjustment for imbalanced baseline factors, including the presence of symptoms other than low back pain, will be performed as supplementary analyses. We will perform a per-protocol analysis as a secondary analysis, defining compliance to the protocol as taking ≥ 80% of the planned number of pills, which is consistent with methodology from a previous trial [7]. Analysis of the moderating effect of Modic change status will use regression models with an interaction term between intervention arm and Modic change status, and adjusting for baseline values of the outcome variables. Missing outcome data for all analyses will be imputed via multiple imputation methods using relevant baseline and post-baseline measurements.

### Economic analysis

An economic evaluation will be performed in conjunction with the clinical trial to investigate the cost-effectiveness and cost-utility of amoxicillin-clavulanate compared with placebo. The economic evaluation will be conducted from a societal and health care perspective and all relevant costs will be included. Costs of the antibiotic treatment will be included, along with costs of other health care utilisation, such as other prescription medication, care by general practitioners, allied health professionals and medical specialists, hospitalisation and professional home-care. Costs for patients and their family will also be measured. Finally, costs of productivity loss due to paid and unpaid work will also be included. Pain will be primary outcome measures in the cost-effectiveness analysis. For the cost-utility analysis, utilities will be expressed as quality-adjusted life years (QALYs) as measured using the EQ-5D-5L.

The mean differences and 95% confidence intervals for total costs, costs of health care utilisation and costs of productivity loss between the antibiotic and placebo groups will be obtained by bias-corrected and accelerated bootstrapping using 5000 replications. Cost-effectiveness ratios will be estimated by dividing the difference in the total costs between the antibiotic group and the placebo group by the difference in the mean effects for pain intensity and disability. A cost-utility ratio will be determined for QALYs gained over a 12-month follow-up. To determine these ratios and uncertainty surrounding the ratios, the bias-corrected percentile bootstrapping method (5000 replications) will be applied and will be graphically presented on cost-effectiveness planes. Acceptability curves will also be used to determine the probability that the antibiotic therapy was cost-effective compared with placebo at different values of the maximum acceptable ratio.

### Data integrity and management

All collected data will be recorded using case report forms or questionnaires and stored in a locked area in the Department of Epidemiology and Preventive Medicine at Monash University with secured and restricted access. The electronic data will be stored in a password-protected database with secured and restricted access. All data collected will be kept strictly confidential. Data transfer will be encrypted with all data de-identified. Only research personnel on the project will have access to the study data. The trial will be audited through the Monash University School of Public Health and Preventive Medicine and the Alfred Hospital Human Research Ethics Committee.

### Withdrawal

If participants withdraw before completion of the study, the reason and date will be recorded and participants will be asked if they can complete the remaining outcome measures.

### Monitoring

The principal investigators will monitor the conduct and progress of the project and ensure that all trial procedures are compliant with the trial protocol. The research team will have regular meetings to ensure efficient study execution and ongoing monitoring of adverse events. Any changes in the study protocol or procedures will be first approved by the Alfred Hospital Human Research Ethics Committee. Deviations from the original protocol will be fully documented using a breach report form and the protocol will be updated on the Australian New Zealand Clinical Trials Registry. The trial will be formally stopped if metformin is found to have unacceptable side effects.

## Discussion

We present the protocol for a double-blind, randomised, placebo-controlled trial investigating whether antibiotic treatment (amoxicillin-clavulanate) is effective in the management of chronic low back pain with disc herniation and if the presence of Modic changes predicts those individuals more likely to respond to antibiotic therapy. If antibiotic treatment is found to be effective, it will provide high-quality evidence to support this therapeutic approach for a targeted patient subgroup with chronic low back pain and disc herniation. However, if it is not effective, it will provide important data to prevent the inappropriate use of antibiotics in this patient population.

The current trial was designed to examine the efficacy of antibiotic treatment for low back pain with disc herniation, in conjunction with considering the study methodology and results of previous clinical trials. The clinical trials of Albert et al. [6] and Bråten et al. [7] differ in several ways in their methodologies (Table 2). With respect to the choice of antibiotic treatment, Albert et al. investigated the efficacy of the antibiotic, amoxicillin-clavulanate [6], based on the results of a previous biopsy study which found several different types of bacteria to be present in the spinal discs of people with low back pain [24], as well as evidence that the amoxicillin-clavulanate can enter the lumbar disc [13]. In contrast, the trial by Bråten et al. examined the efficacy of the antibiotic, amoxicillin [7]. Amoxicillin-clavulanate is not available in Norway where the study was undertaken. The investigators suggest that the use of amoxicillin with clavulanic acid may increase the risk of side effects and may not be required as culture studies in Europe indicate little or no resistance to *Cutibacterium acnes* [25].

**Table 2:** Comparison of clinical trial methodologies

Clinical trials/protocol	Albert et al. [6]	Bråten et al. [7]	Urquhart et al. (2021)
Patient subgroup			
	Participants with disc herniation and type 1 Modic changes	Participants with disc herniation and type 1 and 2 Modic changes	Participants with disc herniation—with and without type 1 and 2 Modic changes
Intervention			
	Amoxicillin-clavulanate (500/125 mg) 100 days	Amoxicillin (750 mg) 3 months	Amoxicillin-clavulanate (500/125 mg) 3 months
Other treatments allowed			
	No other treatment allowed during the 1 year trial period.	Usual care allowed Encouraged to limit non-steroidal anti-inflammatories	Usual care allowed Use of non-opioid analgesia and non-steroidal anti-inflammatories permitted

We have chosen to examine the efficacy of amoxicillin-clavulanate in our clinical trial as it is a broad-spectrum antibiotic, with clavulanic acid also having an antibacterial effect. This is based on our systematic review, which found that of the 9 studies that examined spinal disc material, 8 reported the presence of more than one bacteria, and *Cutibacterium acnes* and *CN staphylococci* were found to be the most prevalent [8]. Given the question of whether antibiotics are effective for low back pain remains unanswered and amoxicillin-clavulanate was used in the study by Albert et al. and found to have a beneficial effect [6], we wanted to avoid the potential limitation that our antibiotic coverage was inadequate. Moreover, while there is potential for side effects, in particular liver injury, with the use of amoxicillin-clavulanate, the risk is extremely low (21 cases of liver failure in 28 years) [26] and will be significantly minimised in our trial by excluding those with a risk factor for liver injury and ensuring regular monitoring with blood tests during the treatment period. In addition, we chose to investigate the efficacy of amoxicillin-clavulanate for a 3-month period, as the study by Albert et al. used amoxicillin-clavulanate for 100 days and found a beneficial effect [6], and our patient subgroup is considered to be similar to a low-grade vertebral osteomyelitis, which is commonly managed with antibiotic therapy for similar treatment durations (4–6 weeks to 6 months).

There has been variation between the clinical trials in the additional care participants are allowed to access. While participants in the Albert et al. trial were not allowed to seek other treatments during the 1-year trial period [6], those in the Bråten et al. trial were permitted to continue ongoing treatment, but recommended not to start additional treatments or use non-steroidal anti-inflammatory drugs [7]. We have chosen to allow participants in both groups to seek usual care, including the use of analgesia and non-steroid anti-inflammatory medication. We do not expect usual care to affect the proposed mechanism of the antibiotic therapy that is being tested in this trial and usual care to be balanced across the treatment groups due to randomisation. All care provided to each individual patient in both groups will be documented.

The previous clinical trials of antibiotics both targeted patient subgroups with disc herniation and Modic changes, but with Albert et al. recruiting patients with type 1 Modic changes only [6] and Bråten et al. including those with type 1 or 2 Modic changes [7]. It is hypothesised that Modic changes result from a bacterial infection in the disc with cytokine and propionic acid production causing inflammation in the adjacent bone. While a biopsy study by Albert et al. has shown that 80% of individuals with infected disc material developed new Modic changes [24], it is unclear whether Modic changes are important in patient selection. In the biopsy study, low virulent bacteria were also identified in participants who did not develop new Modic changes, and conversely, those that had no bacteria present also developed Modic changes [24]. Moreover, our systematic review found that disc herniation was the most common diagnosis in those individuals with back pain and bacterial infection, and there was a relationship between the presence of bacteria and low back pain with disc herniation [8]. Based on these results, our trial will examine the efficacy of antibiotic treatment in individuals with chronic low back pain and disc herniation confirmed on MRI. There is a potential with restricting our criteria to those with Modic changes that we could miss a subgroup of patients that may benefit from antibiotics. However, to determine the role of Modic changes, we will include people with and without Modic changes and determine whether the presence of Modic changes predicts those individuals more likely to respond to antibiotic treatment.

This trial has several potential limitations that need to be considered. Given we are examining the effectiveness of amoxicillin-clavulanate, which is a broad-spectrum antibiotic, it is not possible to extrapolate the findings to other types of antibiotics. The potential for adverse events is an important consideration with the use of amoxicillin-clavulanate for a 3-month period. We will record the type, frequency and severity of adverse events that occur during the trial. However, the current study is not designed to comprehensively evaluate adverse events; to determine this, prospective cohort studies with larger sample sizes are needed. Antibiotic resistance is an important issue, which is highlighted by the World Health Organisation’s statement that antimicrobial resistance is a global threat to health which needs urgent action at the highest political level [27, 28]. While there is potential for benefit for individuals with low back pain, the potential for harm with the inappropriate use of antibiotics is also significant. If there is growing evidence to show the effectiveness of antibiotic treatment for the management of back pain, investigation in future trials is paramount.

Low back pain is a major global health problem [1], and there is an urgent need for effective, evidence-based treatments [2]. While there is huge interest in the potential effectiveness of antibiotic treatment for chronic low back pain with disc herniation, two clinical trials have reported conflicting results [6, 7]. Thus, the efficacy of antibiotic therapy for chronic low back pain and the patient subgroup that may benefit is still unknown. This clinical trial will provide high-quality evidence to determine whether amoxicillin-clavulanate is effective for the management of low back pain with disc herniation. If amoxicillin-clavulanate is found to be effective, it could be used to reduce pain and disability in individuals with chronic low back pain and, in turn, reduce the huge burden associated with this condition.

## Trial status

The study protocol version number and date is version 4 16 March 2018. Recruitment began on 14 September 2015, and we have randomised 166 participants to date. We randomised all participants by 27 May 2021, and the 12-month outcomes will be completed by 27 May 2022.

## Supplementary Information

**Additional file 1.** SPIRIT 2013 Checklist: Recommended items to address in a clinical trial protocol and related documents

## Data Availability

The final datasets will only be accessed by investigators and research teams with ethical approval. The datasets used or analysed during the current study will be available from the corresponding author following completion of the study analysis and on reasonable request.

## Abbreviations

CRP: c-reactive protein
EQ-5D-5L: EuroQol Instrument
FBE: Full blood examination
MRI: Magnetic resonance imaging
CONSORT: Consolidated Standards of Reporting Trials guidelines
SPIRIT: Standard Protocol Items: Recommendations for Interventional Trials guidelines
QALYs: Quality-adjusted life years
